# The combination of a sphingosine kinase 2 inhibitor (ABC294640) and a Bcl‐2 inhibitor (ABT‐199) displays synergistic anti‐myeloma effects in myeloma cells without a t(11;14) translocation

**DOI:** 10.1002/cam4.1543

**Published:** 2018-05-15

**Authors:** Pasupathi Sundaramoorthy, Cristina Gasparetto, Yubin Kang

**Affiliations:** ^1^ Division of Hematological Malignancies and Cellular Therapy Duke University Medical Center Durham NC USA

**Keywords:** Bcl‐2 inhibitor, multiple myeloma, SK2 inhibitor, sphingosine kinase 2

## Abstract

Multiple myeloma (MM) remains an incurable disease in need of the development of novel therapeutic agents and drug combinations. ABT‐199 is a specific Bcl‐2 inhibitor in clinical trials for MM; however, its activity as a single agent was limited to myeloma patients with the t(11;14) translocation who acquire resistance due to co‐expression of Mcl‐1 and Bcl‐xL. These limitations preclude its use in a broader patient population. We have recently found that a sphingosine kinase 2‐specific inhibitor (ABC294640) induces apoptosis in primary human CD138^+^ cells and MM cell lines. ABC294640 is currently in phase I/II clinical trials for myeloma (clinicaltrials.gov: #NCT01410981). Interestingly, ABC294640 down‐regulates c‐Myc and Mcl‐1, but does not have any effects on Bcl‐2. We first evaluated the combinatorial anti‐myeloma effect of ABC294640 and ABT‐199 in vitro in 7 MM cell lines, all of which harbor no t(11;14) translocation. Combination index calculation demonstrated a synergistic anti‐myeloma effect of the combination of ABC294640 and ABT‐199. This synergistic anti‐myeloma effect was maintained even in the presence of bone marrow (BM) stromal cells. The combination of ABC294640 and ABT‐199 led to enhanced cleavage of PARP and caspase‐3/9 and increased Annexin‐V expression, consistent with the induction of apoptosis by the combination treatment. In addition, the combination of ABC294640 and ABT‐199 resulted in the down‐regulation of the anti‐apoptotic proteins Mcl‐1, Bcl‐2, and Bcl‐xL and the cleavage of Bax and Bid. The combination induced both the mitochondrial mediated‐ and caspase‐mediated apoptosis pathways. Finally, the combination of ABC294640 and ABT‐199 resulted in augmented anti‐myeloma effect in vivo in a mouse xenograft model. These findings demonstrate that the co‐administration of ABC294640 and ABT‐199 exhibits synergistic anti‐myeloma activity in vitro and in vivo, providing justification for a clinical study of this novel combination in patients with relapsed/refractory multiple myeloma.

## 1 INTRODUCTION

Multiple myeloma (MM) is a malignant plasma cell disorder with no standard curative therapy.^1^ MM affects 4.3 per 100 000 individuals yearly,^2^ and accounts for about 1% of all cancers and 10% of all hematologic malignancies in the United States.^3^ Over the last 2‐3 decades, the outcomes and survival of patients with MM have significantly improved largely due to the use of “biologic” agents—immunomodulatory drugs, proteasome inhibitors, and monoclonal antibodies, and the incorporation of autologous hematopoietic stem‐cell transplantation. However, despite these advances in treatment, MM remains an incurable disease. Patients may relapse within months after autologous hematopoietic stem‐cell transplantation. Furthermore, nearly all MM patients will eventually develop resistance to currently available agents. There is an unmet medical need for the development of novel therapeutic agents and/or novel drug combinations for MM. It is particularly important to develop new agents or drug combinations that do not share similar mechanisms of action with proteasome inhibitors or immunomodulatory drugs because most of the refractory/relapsed MM patients have been exposed to those agents during their course of treatment.

Sphingolipids are an extremely diverse group of water‐insoluble molecules that include ceramides, sphingoid bases, ceramide phosphates, and sphingoid base phosphates.^4^ In addition to supporting the structure and fluidity of the lipid bilayer, sphingolipid metabolites function as second messengers, and regulate cytokine‐mediated cell signaling.^5^, ^6^ Sphingolipids are involved in a wide range of biologic and pathologic events including inflammation, cell proliferation, apoptosis, angiogenesis, and transformation (reviewed in ref. 7, 8, 9, 10, 11, 12). Sphingolipid metabolism is increasingly recognized as a key pathway in tumor cell survival and in cancer biology.^13^, ^14^, ^15^, ^16^, ^17^, ^18^, ^19^, ^20^

Sphingosine kinases 1 and 2 (SK1 and SK2) phosphorylate sphingosine to sphingosine‐1‐phosphate (S1P). Ceramide and sphingosine are pro‐apoptotic, inducing apoptosis in tumor cells without disrupting quiescent normal cells.^21^, ^22^, ^23^, ^24^ In contrast, S1P is mitogenic and anti‐apoptotic. A critical balance (ie, a ceramide: S1P rheostat) is hypothesized to determine the fate of the cell.^14^, ^25^, ^26^ SK2 is an innovative molecular target for anti‐cancer therapy. We have demonstrated that SK2 is overexpressed in MM cell lines and in human MM specimens.^4^ ABC294640 (YELIVA^®^) [3‐(4‐chlorophenyl)‐adamantane‐1‐carboxylic acid (pyridin‐4‐ylmethyl) amide, hydrochloride salt] is an orally available inhibitor of SK2.^4^ Inhibition of SK2 by RNA interference or treatment with ABC294640 effectively promotes apoptosis in MM cell lines and inhibits the proliferation of primary human myeloma cells. Furthermore, ABC294640 up‐regulates Noxa expression and reduces the expression of Mcl‐1 and c‐Myc by inducing their proteasome degradation.^4^ ABC294640 effectively inhibited myeloma tumor growth in vivo in mouse xenograft models, and has recently completed a Phase I safety study where it demonstrated an excellent safety profile in solid tumors.^27^ Our group has recently started a phase I/II clinical trial with single agent ABC294640 in relapsed and refractory multiple myeloma patients (clinicaltrials.gov: #NCT01410981).

The Bcl‐2 family proteins are key regulators of the intrinsic (mitochondrial) apoptotic pathway and consist of pro‐ and anti‐apoptotic proteins. The anti‐apoptotic proteins (Bcl‐2, Bcl‐xL, and Mcl‐1) bind with pro‐apoptotic proteins (Bim, Bid, Bak, and Bax), thereby sequestering the pro‐apoptotic proteins in a neutralized state and preventing them from inducing apoptosis. ABT‐199 (Venetoclax^®^) is a novel, orally bioavailable small molecule inhibitor of Bcl‐2 with a much lower affinity for other anti‐apoptotic proteins Bcl‐xL and Bcl‐W (>480‐fold and >2,000‐fold lower affinity, respectively).^28^, ^29^, ^30^, ^31^ ABT‐199 was developed to specifically target Bcl‐2 and spare Bcl‐xL and Mcl‐1, thus preventing deleterious adverse effects such as thrombocytopenia and T‐cell lymphopenia seen with previous generations of Bcl‐2 inhibitors.^32^, ^33^, ^34^, ^35^, ^36^ ABT‐199 has demonstrated activity against B‐cell follicular lymphomas, mantle cell lymphomas, diffuse large B‐cell lymphomas, acute myeloid leukemia, and MM. Treatment of relapsed/refractory MM patients with single agent ABT‐199 revealed an acceptable safety profile and evidence of activity in patients; especially those with a t(11;14) translocation and those with a favorable Bcl‐2 family profile.^33^ MM cell lines and primary myeloma cells bearing the t(11;14) translocation were particularly sensitive to ABT‐199. The sensitivity of MM cell lines to ABT‐199 correlated most closely with their Bcl‐2/Mcl‐1 mRNA expression ratio, with the most sensitive cell lines expressing high levels of Bcl‐2 relative to Mcl‐1, a known resistance factor for Bcl‐2 inhibitors.

ABT‐199 activity relies on t(11;14), high Bcl‐2/Mcl‐1 or high Bcl‐2/Bcl‐xL ratio. Translocation (11;14) results in a dysregulated cyclin D1 expression under the transcription control of immunoglobulin heavy chain and an accelerated G1 to S phase transition.^37^ Patients with a t(11;14) translocation only account for 15‐20% of all myeloma patients,^38^, ^39^, ^40^ and single agent ABT‐199 has very limited activity on myeloma cells without the translocation. Furthermore, ABT‐199 has no effects on Mcl‐1, and up‐regulation of Mcl‐1 is one of the main molecular mechanisms contributing to ABT‐199 resistance. ABT‐199 binds to Bcl‐2, releasing the pro‐apoptotic protein Bim. However, in the presence of Mcl‐1 and Bcl‐xL, Bim can bind to Mcl‐1 and/or Bcl‐xL and is unable to cause apoptosis, rendering resistance to ABT‐199. Resistance to ABT‐199 occurs very quickly resulting in rapid disease relapse.^41^, ^42^ Overexpression of Mcl‐1 has been observed in various cancers including MM and plays a major role in myeloma cell survival and resistance to chemotherapy.^43^, ^44^ Due to these limitations, there has been great interest in combining ABT‐199 with other agents to augment ABT‐199's activity in patients without a t(11;14) translocation, and to prevent drug resistance to ABT‐199.

ABC294640 induces proteasome degradation and down‐regulation of Mcl‐1 and c‐Myc, but has no effect on Bcl‐2. On the other hand, ABT‐199 inhibits Bcl‐2, but has no effect on Mcl‐1 and Bcl‐xL. We hypothesize that the combination of ABC294640 and ABT‐199 will lead to synergistic anti‐myeloma effects. In the current study, we investigated the anti‐myeloma effects of ABC294640 combined with ABT‐199. Our results show that the combination of ABC294640 and ABT‐199 synergistically induced apoptosis in MM cells in vitro and in vivo. Our findings suggest a role for the combination of ABT‐199 and ABC294640 in MM patients.

## 2 MATERIALS AND METHODS

### 2.1 Cell culture

Human multiple myeloma cell lines (AMO1, JJN3, L363, and OPM2) were kindly provided by Dr. Bernard Klein at Institute of Human Genetics, Montpellier, France. Human myeloma cell lines (RPMI8226, RPMI8226/DOX, and U266) were originally purchased from the American Type Culture Collection (Manassa, VA, USA). All MM cell lines were grown in RPMI1640 medium supplemented with 10% fetal bovine serum and 1% penicillin/streptomycin at 37°C with 5% CO_2_ in a humidified incubator.

### 2.2 Reagents and antibodies

ABC294640 (the SK2‐specific inhibitor) was synthesized and provided by Apogee Biotechnology Corp (Hummelstown, PA, USA). Bcl‐2 inhibitor (ABT‐199) was purchased from Selleckchem (Houston, TX). Primary antibodies against poly(ADP‐ribose) polymerase‐1, caspase‐3, caspase‐9, Bim, Bad, Bax, Bid, Bcl‐2, Bcl‐xL, Mcl‐1, c‐Myc, and GAPDH, and the corresponding secondary antibodies for western blotting were purchased from Cell Signaling Technology (Danvers, Massachusetts, USA). Propidium iodide, ribonuclease, and 3‐(4,5‐dimethylthiazol‐2‐yl)‐2,5‐diphenyl tetrazolium bromide (MTT) and dimethyl sulfoxide (DMSO) were obtained from Sigma‐Aldrich (St. Louis, MO, USA).

### 2.3 MTT assay

The cytotoxicity of ABC294640 and ABT‐199 was determined by MTT assay. ABC294640 and ABT‐199 were dissolved in DMSO at 60 mmol/L and 10 mmol/L, respectively, and the final concentration of DMSO was 0.1% in the vehicle control for all the experiments. Briefly, cells were treated with various concentrations of ABC294640 and/or ABT‐199 for 48 hours. MTT (5 mg/mL in PBS, 20 μL) dye was added and incubated for 2 hours at 37°C in a humidified incubator containing 5% CO_2_. The media was removed and cells were then pelleted, and insoluble formazan complexes were solubilized with 100 μL of DMSO and the absorbance was measured at 570 nm using microplate spectrophotometer (Bio‐Rad). MM cell survival and the combination index (CI) were calculated using CalcuSyn software (Biosoft). All experiments were conducted in triplicate and repeated at least twice.

### 2.4 Western blot

Western blotting was performed as described previously.^45^, ^46^ Briefly, cells were collected and lysed with RIPA buffer (Thermo Scientific, MA, USA). Approximately, 20 μg protein was loaded and run on sodium dodecyl sulfate‐polyacrylamide gel electrophoresis (SDS‐PAGE). The proteins were transferred onto polyvinylidene difluoride membrane (Millipore). The membrane was blocked with 5% skim milk and subsequently incubated with the specific primary antibodies overnight at 4°C with gentle shaking. The membrane was then probed with ECL system (AbClon) for signal detection. Films were developed using a Kodak M35‐A X‐OMAT processor.

### 2.5 Annexin V and propidium iodide staining

JJN3, OPM2, and RPMI8226 cells were seeded on 6‐well plates (2 × 10^5^ cell/mL) and treated with ABC294640 and/or ABT‐199. Apoptosis was determined by the Annexin‐V FITC Apoptosis Detection Kit, which was performed according to the manufacturer's instructions (BD Pharmingen). Data were acquired on a FACSCalibur flow cytometer (BD Biosciences). Results were obtained by analyzing data with FlowJo software.

### 2.6 Mitochondrial membrane potential (ΔΨm) assay

Cells were seeded into 6‐well plates and treated with ABC294640 and/or ABT‐199 for 12 hours. After treatment, cells were collected by centrifugation at 300 × *g* at 4°C for 5 minutes. Mitochondrial outer membrane potential was assessed by JC‐1 staining (5,5′,6,6′‐tetrachloro‐1,1′,3,3′‐tetraethyl‐benzimidazolylcarbocyanin iodide), which was performed according to the manufacturer's instructions (Life Technologies). JC‐1 is a cationic dye that forms a red fluorescent J‐aggregate in mitochondria with a high membrane potential and low membrane potential JC‐1 shows a cytosolic green fluorescence. Mitochondrial membrane potential was analyzed by flow cytometry and cells having increased green fluorescence were assumed as cells with reduced mitochondrial membrane potential (ΔΨm).

### 2.7 In vivo myeloma xenograft mouse experiment

The mouse study was conducted in accordance with guidelines approved by the Institutional Animal Care and Use Committees at Duke University. JJN3 cells (3‐5 × 10^5^ cells/mouse) were injected subcutaneously into sub‐lethally irradiated (1.5 Gy) non‐obese diabetic severe combined immunodeficiency IL‐2λ null mice (NSG) mice (8‐week old; Jackson Laboratory). When the tumor became measurable, animals were randomly divided into 4 groups (n = 5 per group): control group, ABC294640, ABT‐199, and the combination of ABC294640 and ABT‐199. The drugs were dissolved in 60:30:10 (PEG400: Phosal G: Ethanol) and were orally administered every day for 20 days at a dose of 50 mg/kg/d for ABC294640 and ABT‐199. Tumor size was measured twice a week using a digital caliper and the tumor volume (*V*) was calculated as *V* = ((width)^2^ × length)/2. The animals were sacrificed at the end of experiment and the primary tumor was excised, weighed, and subjected to further analysis.

### 2.8 Statistical analysis

All the data were presented as the mean ± SD. Comparison was performed by the student's *t* test for analysis of variance for continuous data. All statistical analyses were performed using Star View software (SAS institute, Cary, NC) or Microsoft Excel (Microsoft, Seattle, WA). *P* values less than .05 were considered significant.

## 3 RESULTS

### 3.1 The combination of sphingosine kinase 2 inhibitor (ABC294640) and Bcl‐2 inhibitor (ABT‐199) synergistically inhibits myeloma cell growth in vitro

**Figure 1:**
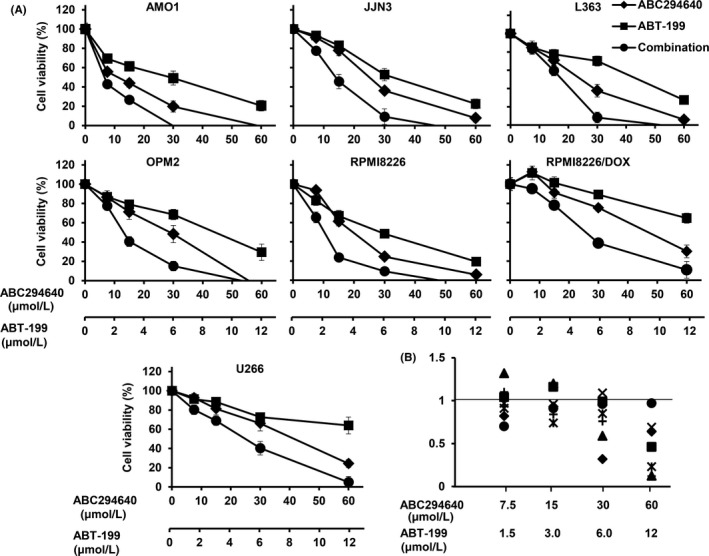
In vitro synergistic effect of ABC294640 and ABT‐199 combination in MM cells. A, AMO1, JJN3, L363, OPM2, RPMI8226, RPMI8226/DOX, and U266 cells were plated into 96‐well plate (3‐5 × 10^5^ cell/well), and treated with various concentration of ABC294640 and ABT‐199, alone or in combination for 48 h followed by MTT assay. B, The combination index was calculated using CalcuSyn software for AMO1 (◆), JJN3 (■), L363 (▲), OPM2 (×), RPMI8226 (✳), RPMI8226/DOX (●), and U266 (+) cell lines. The data were obtained from three independent experiments and is presented as mean ±SD.

In our previous study, we showed that the SK2 inhibitor (ABC294640) decreased multiple myeloma cell proliferation in vitro and exhibited significant anti‐myeloma efficacy in vivo.^4^ In the present study, we investigated the combinatorial effects of ABC294640 and Bcl‐2 inhibitor (ABT‐199) on MM cells. Single agent ABT‐199's anti‐myeloma activity was limited to myeloma cells harboring a t(11;14) translocation. Myeloma cells that do not have a t(11;14) translocation are generally resistant to ABT‐199. To test if combining ABT‐199 with ABC294640 would sensitize myeloma cells without the translocation) to ABT‐199, we treated 7 different myeloma cell lines (AMO1, JJN3, L363, OPM2, RPMI8226, RPMI8226/DOX, and U266) with various concentrations of ABC294640 or ABT‐199 alone or in combination, and measured cell survival using a MTT assay. None of these 7 myeloma cell lines harbor the t(11;14) translocation. Consistent with previous reports, these cell lines were relatively resistant to single agent ABT‐199 with IC_50_s of >30 μmol/L.^4^, ^42^ The addition of ABC294640 led to significantly enhanced anti‐myeloma effects (Figure 1A). To determine if the combinatorial effect is additive or synergistic, we calculated the combination index (CI) using the CompuSyn software. A CI of less than 1 is considered to be a synergetic effect, whereas a CI of >1 is additive. As shown in Figure 1B, the combination of ABC294640 at 15 μmol/L and ABT‐199 at 3 μmol/L demonstrated a synergistic cytotoxic effect in all MM cell lines tested (CI < 1), except L363 cell lines (CI > 1). These data indicate that ABC294640 is able to synergize with ABT‐199 to inhibit the proliferation of MM in a synergistic manner.

### 3.2 The combination of ABC294640 and ABT‐199 demonstrates synergy in inducing apoptotic cell death in myeloma cells

**Figure 2:**
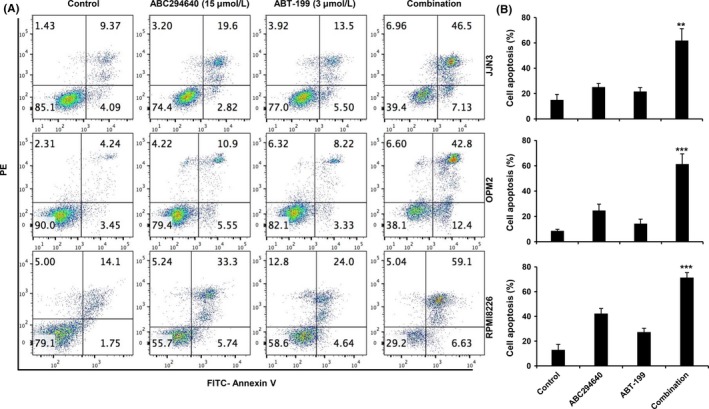
ABC294640 in combination with ABT‐199 induces synergistic apoptosis in MM cells. A, JJN3, OPM2, and RPMI8266 cells treated with ABC294640 (15 μmol/L) and ABT‐199 (3 μmol/L), either alone or in combination for 16 h, followed by Annexin V‐FITC/PI staining and analysis by flow cytometer and percentage of cells is shown within each quadrant. B, Quantification of cell death percentage. Results are representative of three independent experiments as the mean ± SD. The *P* values were calculated between control and combination treatment (*P = .05; **P = .01; and ***P = .001)

Our previous data have demonstrated that treatment with ABC294640 resulted in apoptosis of MM cells. It has also been shown previously that Bcl‐2 inhibitors can induce apoptosis in MM cells. To test if the combination of ABC294640 and ABT‐199 could induce synergy in apoptosis in MM cells, MM (JJN3, OPM2, and RPMI8226) cells were exposed to ABC294640 (15 μmol/L) and ABT‐199 (3 μmol/L), alone or in combination for 16 hours and the number apoptotic cells was measured using Annexin‐V staining by flow cytometry. As shown in Figure 2A,B, single agent ABC294640 and ABT‐199 induced apoptotic cell death in the range of 16%‐40% and 11%‐30%, respectively. The combination treatment significantly increased apoptosis to 60%‐72%.

**Figure 3:**
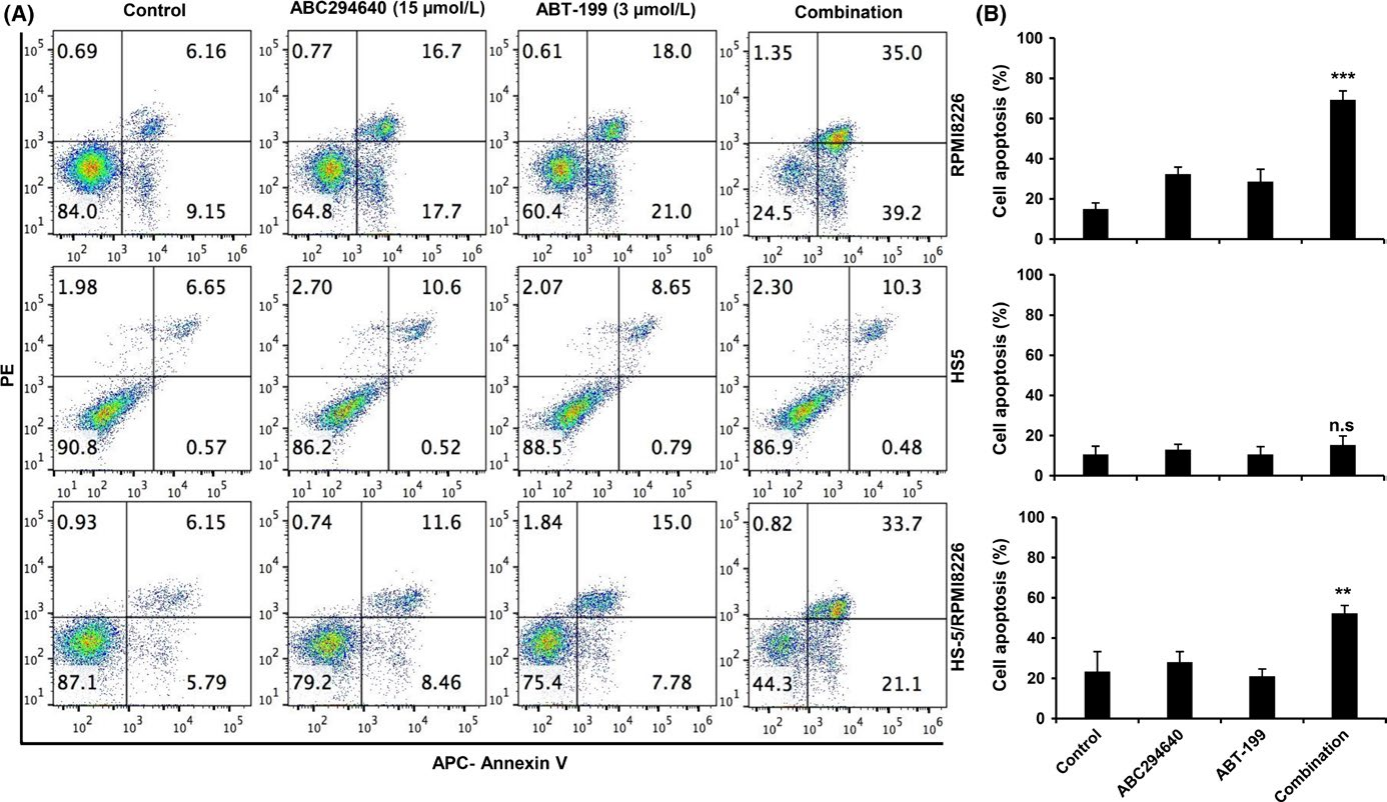
ABC294640 and ABT‐199 synergistically induce apoptosis in MM cells in the presence of BM stromal cells. A, HS5 cells were grown for 48 h to form monolayer, then GFP‐expressing RPM8226 cells were added into the HS5 well for 24 h and subsequently the cells were treated with ABC294640 (15 μmol/L) or ABT‐199 (3 μmol/L), alone or in combination for 24 h. The cells were stained with APC‐Annexin/PI and apoptosis cell were gated on GFP+ cells. B, Quantification of cell death percentage. Results are representative of three independent experiments as the mean ± SD, n.s., no statistical difference. The *P* values were calculated between control and combination treatment (*P = .05; **P = .01; and ***P = .001)

Myeloma cells reside in the bone marrow (BM) and the BM microenvironment plays an important role in MM cell growth, survival, and drug resistance.^47^, ^48^ Thus, we next sought to determine whether the combinatorial effect of ABC294640 and ABT‐199 on myeloma cells still exists when co‐cultured with BM stromal cells. RPMI8226 cells expressing green fluorescent protein (GFP) were co‐cultured with BM stromal HS5 cell line and treated with ABC294640 and ABT‐199 alone or in combination for 24 hours. Apoptosis was measured in GFP^+^ RPMI8226 cells and GFP^−^ HS5 stromal cells (Figure 3A,B). ABC294640, ABT‐199, or the combination failed to induce apoptosis in HS5 stromal cells. On the other hand, ABC294640 and ABT‐199 combination demonstrated synergistic effects in killing RPMI8226 in the presence of HS5 stromal cells, similar to RPMI8226 cells alone. These results indicate that ABC294640 synergistically acts with ABT‐199 in the induction of apoptosis in MM cells, even in the presence of stromal cells.

### 3.3 Combination of ABC294640 and ABT‐199 resulted in augmented PARP cleavage and Caspase 3 and 9 activation

**Figure 4:**
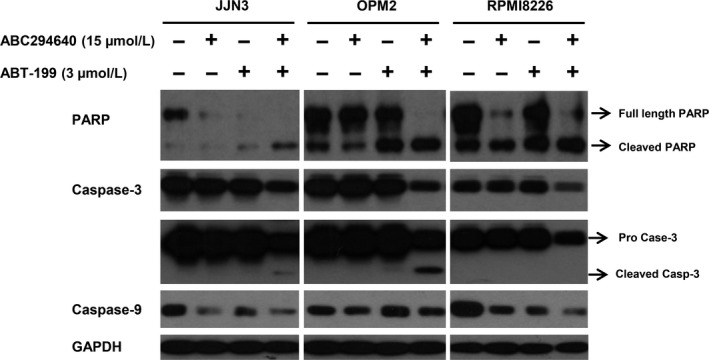
ABC294640 and ABT‐199 combination induces caspase‐mediated apoptosis in MM cells. JJN3, OPM2, and RPMI8266 cells were treated with ABC294640 and ABT‐199 either alone or in combination at indicated concentration for 16 h. Whole cell lysate was subjected for immunoblot analysis with PARP, caspase‐3, ‐9, and GAPDH antibodies. The immunoblots shown here are representative of 2 or 3 experiments

Apoptotic cell death is characterized by the cleavage of poly(ADP‐ribose) polymerase (PARP), and the cleavage and activation of pro‐Caspase 3. Pro‐caspase‐3 is cleaved by caspase‐8, caspase‐9, and granzyme B to form the active heterodimer of caspase‐3 subunits. Caspase‐3 then cleaves PARP into a 89‐Kda and 24‐Kda fragments, which trigger apoptosis signaling pathways.^49^ Caspase activation plays an important role in the activation of apoptosis: caspases can activate the apoptotic signaling pathway and thus lead to cell death.^50^, ^51^ To characterize whether apoptosis was the mode of cell death induced by ABC294640 in combination with ABT‐199, caspase and PARP cleavage was assessed in JJN3, OPM2, and RPMI8226 cells by western blot analysis. As seen in Figure 4, compared to single agent alone, treating the cells with ABC294640 (15 μmol/L) and ABT‐199 (3 μmol/L) in combination for 16 hours resulted in enhanced down‐regulation of pro‐caspase‐3, caspase‐9, and full‐length PARP, with increased cleavage products of caspase‐3 and PARP in all three MM cell lines.

### 3.4 Combination of ABC294640 and ABT‐199 results in enhanced down‐regulation of Mcl‐1 and Bcl‐xL in myeloma cells

**Figure 5:**
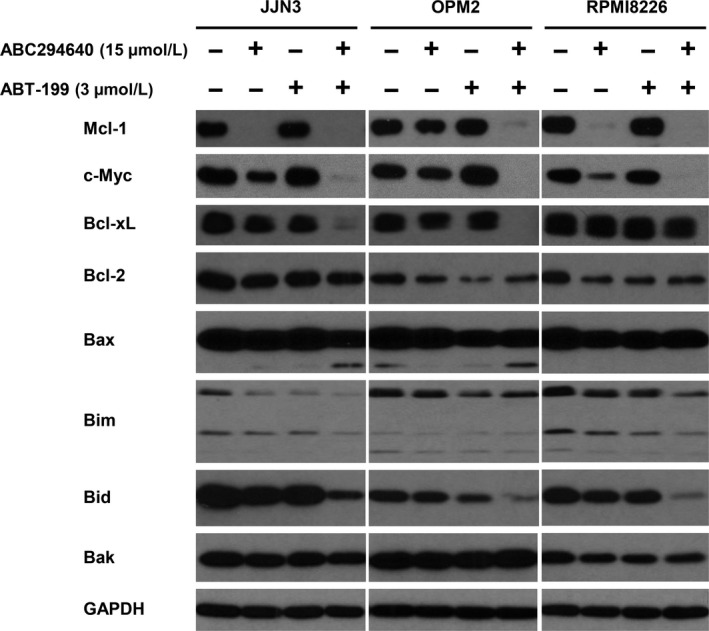
Changes of anti‐apoptotic Bcl‐2 family protein by ABC294640 and ABT‐199 combination in MM cells. JJN3, OPM2, and RPMI8266 cells were treated with indicated concentration for 16 h, either alone or in combination. Anti‐apoptotic proteins Mcl‐1, Bcl‐2, and Bcl‐xL, and pro‐apoptotic proteins Bax, Bim, Bid, and Bak were analyzed by immunoblot. The data shown are representative of 2 or 3 experiments

While ABT‐199 is able to inhibit Bcl‐2, it has no effects on Mcl‐1 and Bcl‐xL. In fact, expression of Mcl‐1 and Bcl‐xL was the underlying mechanism driving drug resistance to ABT‐199 in myeloma cells. Previously, we showed that treatment of ABC294640 led to down‐regulation of Mcl‐1 and c‐Myc expression via proteasome degradation in MM cells.^4^ To analyze the mechanism underlying the synergy of ABC294640 and ABT‐199, we investigated the changes of Bcl‐2 family members after combination treatment. Consistent with our previous results, treatment with ABC294640 decreased the levels of Mcl‐1, c‐Myc, and more importantly Bcl‐xL in all three MM cell lines. On the other hand, ABT‐199 alone did not affect the expression of all these three proteins (Figure 5). When treated with the combination, the expression of Bcl‐xL is further decreased compared to that with ABC294640 treatment alone. Similarly, the levels of Mcl‐1 and c‐Myc expression in the combination treatment were significantly decreased in comparison to those with ABC294640 alone in all three MM cell lines.

**Figure 6:**
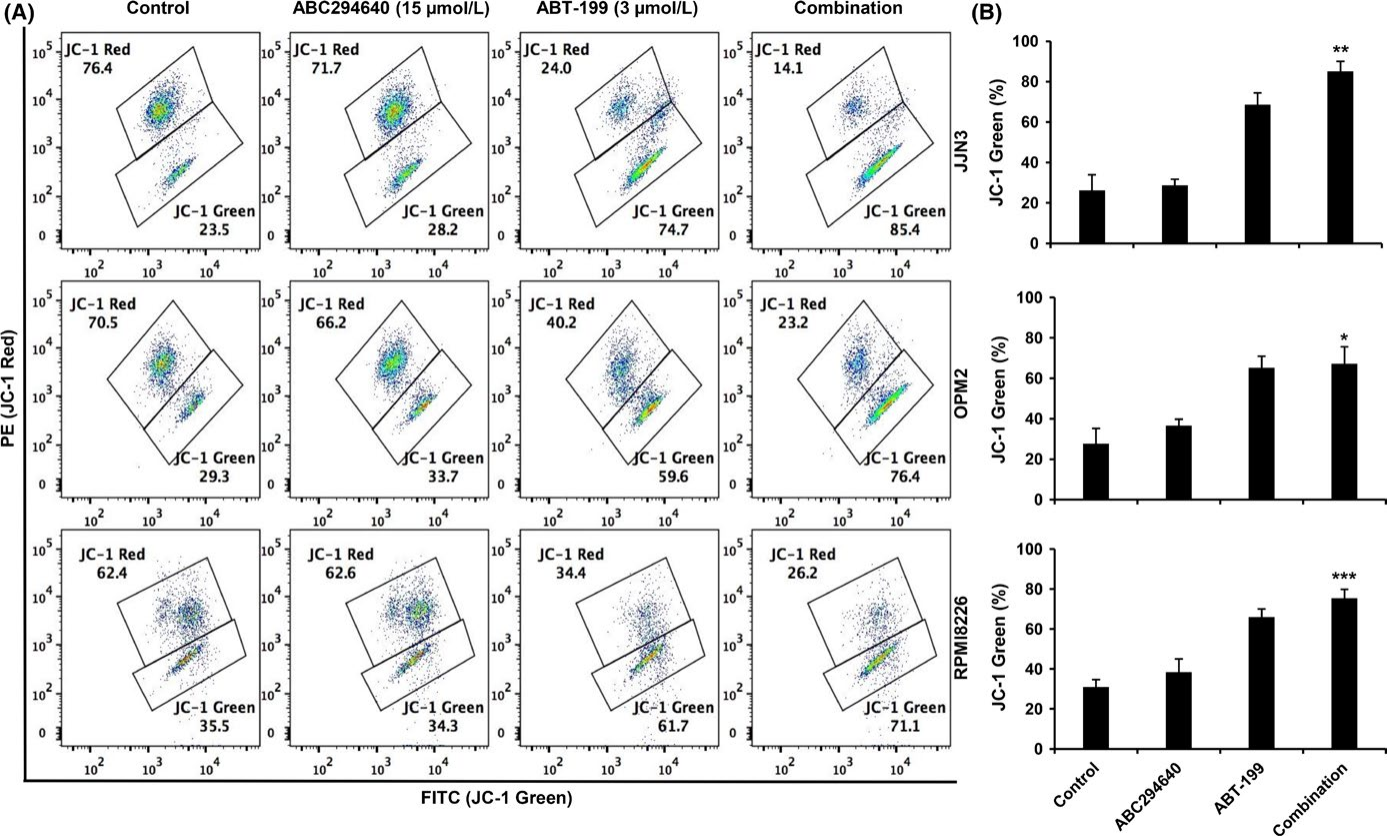
ABC294640 and ABT‐199 synergistically induce mitochondrial membrane damage in MM cells. A, JJN3, OPM2, and RPMI8266 cells were treated with indicated concentration for 16 h, either alone or in combination. An increased green fluorescence indicates that mitochondrial membrane depolarization. B, Quantification of green florescence. Results are representative of three independent experiments as the mean ± SD. The *P* values were calculated between control and combination treatment (*P = .05; **P = .01; and ***P = .001)

The effects of the combination of ABC294640 and ABT‐199 on the expression of pro‐apoptotic proteins were also investigated. As shown in Figure 5, no significant changes were observed in Bak, whereas total Bid was down‐regulated in the combination treatment. Previous studies have shown that activated caspase induced Bid cleavage.^52^, ^53^ In addition, the level of full‐length Bax was reduced, and cleaved Bax was observed with the combination treatment. Bax cleavage was reported to be associated with mitochondrial‐mediated apoptosis.^54^, ^55^ To further investigate mitochondrial damage, we performed a JC‐1 MitoProbe assay. As shown in Figure 6A,B, the combination treatment led to significant mitochondrial depolarization. Interestingly, the mitochondrial damage was predominantly attributed to ABT‐199, and not ABC294640. These data imply that the combination of ABC294640 and ABT‐199 causes apoptotic cell death from 2 non‐overlapping pathways: one from the intrinsic, mitochondrial‐associated apoptosis by ABT‐199, and the other from extrinsic, cytoplasmic‐associated apoptosis by ABC294640. Taken together, our results demonstrated that the combination of ABC294640 and ABT‐199 led to dramatic down‐regulation of Mcl‐1, c‐Myc, and Bcl‐xL, and activation of both intrinsic and extrinsic apoptosis pathways.

### 3.5 The combination of ABC294640 and ABT‐199 demonstrated enhanced anti‐myeloma activity in vivo in a myeloma xenograft mouse model

**Figure 7:**
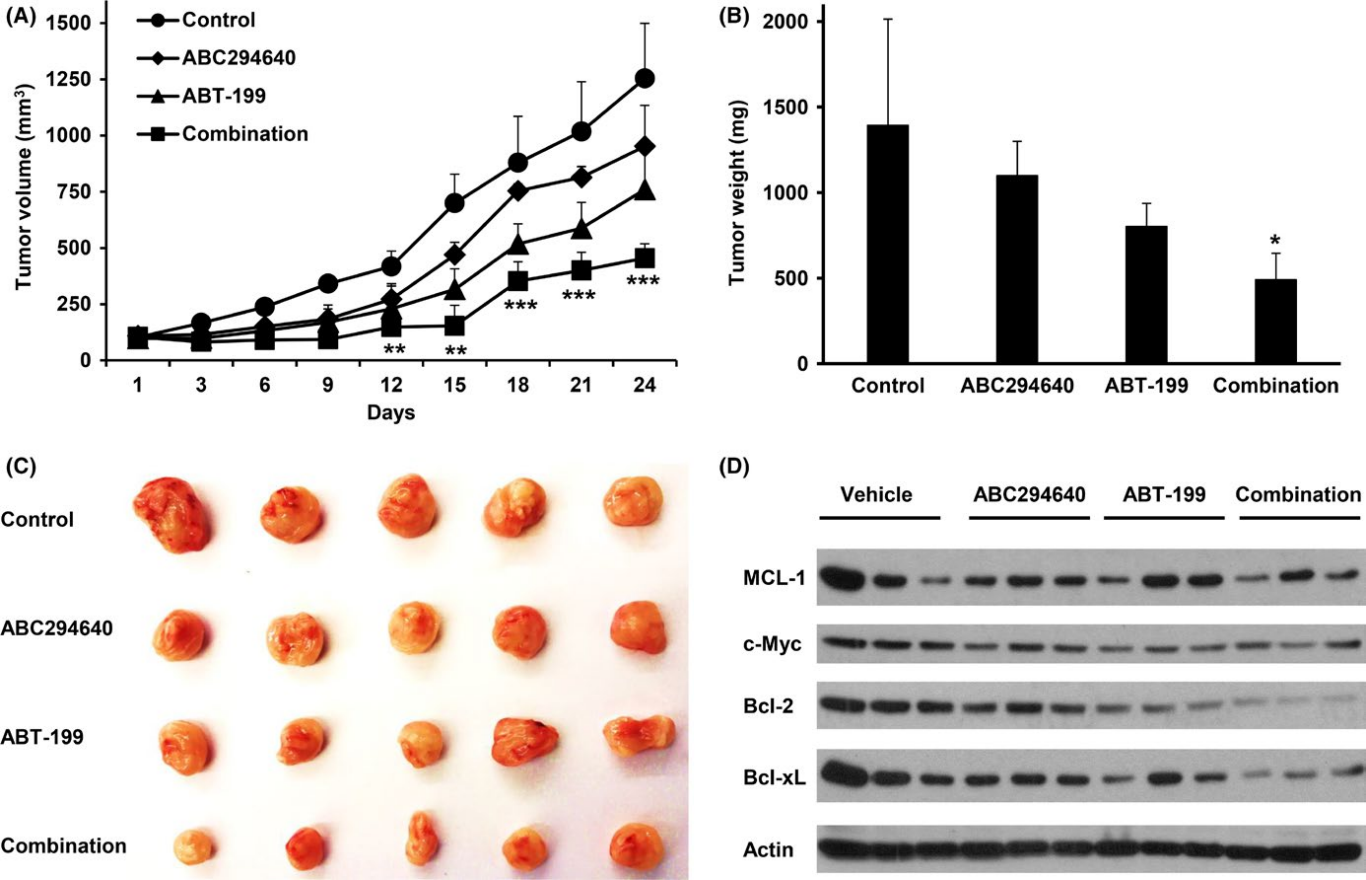
In vivo therapeutic effects of ABC294640 or ABT‐199‐199 combination treatment on established myeloma in NSG mice. A, JJN3 tumors were established in NSG mice, when tumors became measurable the mice were evenly separated into 4 groups (n = 5) with equal tumor size and treated with vehicle, ABC294640 (50 mg/kg) and ABT‐199‐199 (50 mg/kg), alone or in combination every day for 20 d by oral gavage; B, tumor weight; C, tumor image; and D, western blot analysis showed the expression of Mcl‐1, c‐Myc, Bcl‐2, and Bcl‐xl. The *P* values were calculated between control and combination treatment (*P = .05; **P = .01; and ***P = .001)

To determine the in vivo anti‐myeloma activity of the ABC294640 and ABT‐199 combination, we used NSG mice for a xenograft model. JJN3 cells were injected s.c. into sub‐lethally irradiated (1.5 Gy) NSG mice. When the tumor mass became measurable, mice were separated into 4 groups and treated with vehicle, ABC294640 (50 mg/kg), ABT‐199 (50 mg/kg), or in combination, daily, for 20 days by oral gavage. No behavioral changes or weight loss were observed in treated animals. The anti‐tumor effect of ABC294640 or ABT‐199 alone was rather moderate. However, the combination of ABC294640 and ABT‐199 had significantly stronger anti‐tumor effect (Figure 7A‐C). We continued to follow the mice for 5 days after the treatment was stopped. While the tumor size increased in the ABC294640 alone and ABT‐199 alone groups, the tumors remained significantly suppressed in the combination group. Western blots on the tumor samples confirmed that the combination treatment markedly decreased Mcl‐1, c‐Myc, Bcl‐2, and Bcl‐xL (Figure 7D). These findings indicate that co‐administration of ABC294640 and ABT‐199 markedly reduces in vivo myeloma tumor growth associated with the down‐regulation of Mcl‐1, c‐Myc, Bcl‐2, and Bcl‐xL.

## 4 DISCUSSION

Despite advances in chemotherapy and stem‐cell transplantation, multiple myeloma remains an incurable disease. There is unmet medical need to develop novel therapeutic agents and more effective drug combinations. Sphingosine kinase 2 was found to be a novel therapeutic target.^4^ As shown in our previous study, SK2 was overexpressed in MM cell lines and primary human bone marrow CD138^+^ myeloma cells. Inhibition of SK2 by ABC294640 effectively suppressed the myeloma growth in vitro and in vivo.^4^ A phase I/II clinical trial of single agent ABC294640 in relapsed and refractory myeloma patients is currently ongoing (http://www.clinicaltrials.gov: #NCT01410981). In the current study, we examined the combination effect of ABC294640 and Bcl‐2 inhibitor (ABT‐199, venetoclax) on MM cell growth. We found that the combination of the SK2 inhibitor ABC294640 and the Bcl‐2 inhibitor ABT‐199 had a synergistic cytotoxic effect on MM cells in vitro and in vivo.

Induction of apoptosis in tumor cells is an important goal of cancer chemotherapy. It has been shown that anti‐apoptotic members of Bcl‐2 family proteins are crucial regulators of survival in various cancers including MM. Bcl‐2, Bcl‐xL, and Mcl‐1 are significantly overexpressed in myeloma cells, and are considered to be among the major contributing factors to the survival, growth, and chemo‐resistance of myeloma.^32^, ^34^ Therefore, targeting these proteins represents an attractive therapeutic strategy. ABT‐199, a specific Bcl‐2 inhibitor, showed promising therapeutic efficacy in early phase clinical trials in Bcl‐2‐dependent tumor including MM patients with a t(11;14) translocation.^56^ However, ABT‐199 had very limited activity in myeloma patients who did not have the t(11;14) translocation, which accounts for >75% of myeloma patients. Furthermore, studies have shown that expression of Bcl‐xL and/or Mcl‐1 leads to resistance to ABT‐199, and down‐regulating Mcl‐1 and Bcl‐xL could potentially increase the anti‐tumor activity of ABT‐199.^57^, ^58^ These observations suggest that ABT‐199 would best be used in combination therapy.^41^, ^57^ In fact, combination of ABT‐199 with dexamethasone broadens ABT‐199 activity to myeloma cell lines that do not harbor at(11;14) translocation.^57^ Additionally, combination of ABT‐199 and bortezomib demonstrated safety and efficacy in an early phase clinical trial of patients with relapsed myeloma.^59^ Our current study demonstrated synergistic anti‐myeloma effects of ABC294640 and ABT‐199 in vitro and in vivo in myeloma cells that have no t(11;14) translocations. Because ABC294640 does not share the same mechanisms of action as proteasome inhibitors or immunomodulatory agents, ABC294640 and the combination of ABC294640 with ABT‐199 are particularly attractive agents for relapsed and/or refractory myeloma patients who have been previously treated with proteasome inhibitors and immunomodulatory agents. Our study provides justification rational for testing this combination in the clinical setting.

We found that the protein levels of c‐Myc and Mcl‐1 but not Bcl‐2 levels were decreased by ABC294640 treatment in all three MM cell lines, consistent with our previous results, and we found that the down‐regulation of c‐Myc and Mcl‐1 by ABC284640 was through the proteasome degradation pathway.^4^ c‐Myc plays an important role in tumor progression in various cancers and its activation leads to MM survival and progression, whereas c‐Myc inhibition induced cell death indicating that c‐Myc is a promising therapeutic target.^60^, ^61^ In the current study, we showed for the first time that ABC294640 is also able to down‐regulate Bcl‐xL expression, suggesting that ABC294640 effectively increases ABT‐199 anti‐myeloma activity by affecting several Bcl‐2 family members. Consistent with our hypothesis, when the MM cells were treated with the combination of ABC294640 and ABT‐199, all three anti‐apoptotic proteins were significantly decreased. Furthermore, we examined apoptosis‐related proteins such as PARP, pro‐caspase‐3, and, ‐9 and found that the combination treatment increased the cleavage of PARP, pro‐caspase‐3, and ‐9 in all three MM cell lines (Figure 4). We also observed that ABC294640 and ABT‐199 can induce the cleavage of pro‐apoptotic proteins such as Bax (Figure 5). However, the cleavage of Bax does not imply a decrease in pro‐apoptotic function because previous studies have shown that the cleavage of Bax and Bid is mediated by caspase‐dependent processes, and the cleaved Bax and Bid were associated with increased mitochondrial‐mediated apoptotic pathway.^62^, ^63^ To further support this rationale, we performed the mitochondrial membrane potential (MMP) assay in MM cells, and showed ABC294640 does not induce mitochondrial membrane damage, whereas ABT‐199 reduces mitochondrial membrane potential (Figure 6). These data indicate that ABT‐199 acts through mitochondrial‐mediated pathway, whereas ABC294640 induces cell death through cytoplasmic pathway, which could be the reason for the synergistic effect of the ABC294640‐ABT‐199 combination.

Decreased sensitivity to ABT‐199 due to co‐expression or up‐regulation of Mcl‐1 or Bcl‐xL has been previously observed in MM mouse model. In the current study, we aimed at enhancing ABT‐199's anti‐myeloma activity through combination with novel agents. We selected an SK2 inhibitor, which we have previously shown can promote proteasomal degradation of c‐Myc and Mcl‐1 and induce caspase‐mediated apoptosis in MM cell in vitro and in vivo.^4^ In the present study, we showed ABC294640 can also down‐regulate Bcl‐xL in MM cell lines. Importantly, the combination of ABC294640 and ABT‐199 showed statistically significant anti‐myeloma activity (Figure 7).

In summary, our findings show that the combination of ABC294640 and ABT‐199 has synergistic cytotoxicity in MM cells, and that this synergistic effect leads to the induction of apoptosis through regulation of several Bcl‐2 family proteins. Importantly, the experiments carried out in the in vivo model showed significant suppression of myeloma tumor growth with the combination. Taken together, these data provide a novel insight into the potential application of ABC294640 and ABT‐199 for multiple myeloma, and offer support for further clinical evaluation of the combination of ABC294640 and ABT‐199 in patients with multiple myeloma.

## CONFLICT OF INTEREST

None declared.
